# Accuracy, Ease of Use, Safety, and Acceptability of a 23-μL Conical Cup Blood Transfer Device for Use with Rapid Diagnostic Tests

**DOI:** 10.4269/ajtmh.17-0716

**Published:** 2018-07-16

**Authors:** Sandra Incardona, Daniel J. Kyabayinze, David Bell, Bbale Ndawula, Marvis C. Kanyago, Magoma C. Mwancha-Kwasa, Iveth J. González

**Affiliations:** 1 Foundation for Innovative New Diagnostics (FIND), Geneva, Switzerland;; 2 National Malaria Control Division, Ministry of Health, Kampala, Uganda;; 3 Intellectual Ventures, Global Good Fund, Bellevue, Washington;; 4 School of Biomedical Science, Makerere University, Kampala, Uganda;; 5 Women’s Hospital International, Kampala, Uganda;; 6 Health Research and Development Unit, Ministry of Health, Kiambu, Kenya

## Abstract

Devices to safely transfer fixed amounts of finger prick blood to rapid diagnostic tests (RDTs) pose a significant challenge, especially in non-laboratory settings. Following the success of an “inverted cup device” for transfer of 5 μL blood, a prototype with a conical cup shape was developed for transfer of 20 μL blood, the amount needed for human immunodeficiency virus or human African trypanosomiasis (HAT) RDTs. This study determined the volume of blood transferred by this new blood transfer device (BTD) and compared its ease of use, safety, and acceptability with that of a plastic pipette when used by health workers (HWs) for HAT RDTs in northwestern Uganda. After a half-day training, 48 HWs had used the two BTDs with at least 10 patients. The conical cup BTD effectively transferred a mean of 22.76 μL of blood (standard deviation 3.31 μL). A significantly higher proportion of HWs were able to collect the full amount of blood using the conical cup BTD, as compared with the pipette (92.4% versus 74.2%, *P* < 0.001). In HW questionnaires, the conical cup BTD scored higher than the pipette in various aspects of ease of use and safety. In addition, HWs preferred the conical cup BTD (79%), indicating that it was easy to handle, made work faster, and increased their confidence in front of the patient. These findings suggest that the design of the conical cup BTD may be adapted for RDTs requiring 20 μL of blood to facilitate safe and accurate blood transfer.

## INTRODUCTION

Rapid diagnostic tests (RDTs) are increasingly being used in various disease programs, either as a screening tool, as in the case of human African trypanosomiasis (HAT) or as a diagnostic tool, as in the cases of human immunodeficiency virus (HIV) and malaria.^1–4^ There are several benefits of RDT use, especially that large numbers of personnel with minimal training in laboratory techniques can satisfactorily perform and interpret them, offering appropriate diagnosis at point-of-care (POC).^5–7^ To maintain test accuracy and utility while implementing and scaling up RDTs in primary health facilities, in remote areas, and even at the community level, RDTs must be as simple to use and as reliable as possible. Already, issues of false positive and false negative results, faint lines, and other problems have given rise to questions on the reliability of RDTs.^3,5,8–12^ This highlights the importance of conducting good quality training of health workers (HWs) to precisely follow manufacturers’ instructions when performing the RDT.^8,12^

More particularly, reports and anecdotal observation have repeatedly indicated that blood transfer is an aspect of RDT use that poses a significant challenge to many users.^8,13–15^ All commercially available RDT kits are packaged with single-use disposable blood transfer devices (BTDs), which are used to collect, transfer, and deposit a specified amount of blood (ranging from 5 to 50 μL) from a finger prick site to a sample well on the RDT cassette. However, the volume control for some of these devices is not easy, e.g., pipette BTDs require squeezing the bulb and avoiding aspiration of air bubbles, whereas some other RDTs even come with non-calibrated droppers. Concerns about the available BTDs typically fall into three categories: first, the device design may raise the risk of blood exposure (e.g., spillage of blood during the transfer); second, a device may not reliably transfer the correct amount of blood (excess blood can lead to an insufficiently cleared test window, i.e., red background, whereas insufficient blood can lead to false negative test results); and third, the device may be difficult for many HWs to manipulate.^12,15,16^ As more RDTs are developed for various diseases, there also arises the issue that different devices are needed for the transfer of different blood volumes.

**Figure 1.**
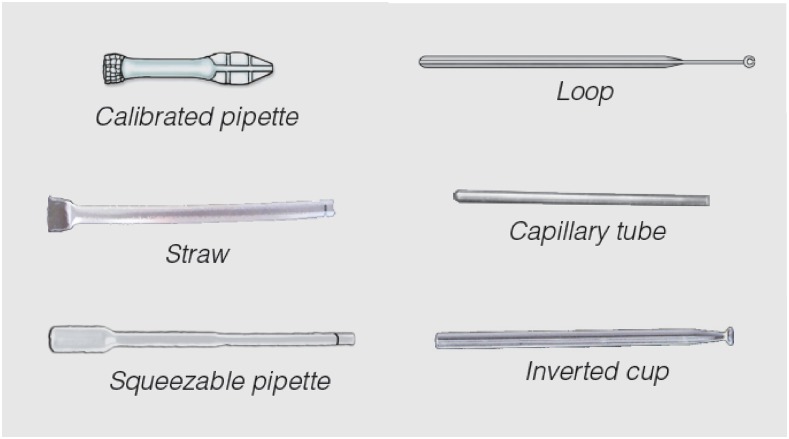
Different types of blood transfer devices included in commercially available malaria rapid diagnostic test kits, and designed for transfer of a fixed blood volume (typically 5 μL). This figure appears in color at www.ajtmh.org.

For the smaller volume transfers, as in malaria RDTs that mostly require 5 μL of blood, there are several BTDs on the market, such as the capillary tube, the straw, the loop, the squeezable or the calibrated pipette and, more recently, the inverted cup transfer device (Figure 1). Some of these allow for blood collection by capillary action, e.g., the loop or the inverted cup device, whereas others require actively controlling the blood volume, e.g., by pressing a bulb in the case of the pipette devices. A study carried out evaluating these various devices showed that the inverted cup device was the most acceptable to HWs in terms of safety, ease of use, and accuracy of volume transferred.^17^ The success of this device led to its subsequent uptake by various manufacturers of malaria RDTs, and at least 170 million inverted cup BTDs were distributed in malaria RDT kits in 2016 (Foundation for Innovative New Diagnostics [FIND], unpublished data), with an increasing trend of distribution volumes observed since the design of this device was made publicly available in 2011. The success of this particular device has been attributed to the easy pick-up of the correct volume of blood by capillary action, and easy deposit by simply touching the filter pad of the RDT.

A number of RDTs for diagnosis of various diseases require higher blood sample volumes. At least 11 brands of HIV RDTs, two brands of hepatitis C virus RDTs, one brand of *Helicobacter pylori* RDTs, and one brand of HAT RDTs require a volume of 20 μL of blood as per the manufacturers’ instructions. Current BTDs available on the market for such a volume were found to be the squeezable plastic pipette, the glass or plastic capillary tube, and the non-calibrated plastic dropper. None of these devices automatically controls blood volume; instead, the user must control the volume by adequate manipulation. To expand on the success of the 5-μL inverted cup BTD and to respond to the need of easier-to-use devices for the transfer of greater blood volumes, a new BTD was designed to transfer approximately 20 μL of blood. After various designs were explored and prototypes tested, the most suitable device—called the “conical cup BTD”—was selected for a field evaluation. The conical cup device is designed to collect a fixed volume of blood through capillary forces, similarly to the 5-μL inverted cup BTD.

This study was designed to determine the accuracy of the conical cup BTD and to evaluate its ease of use, blood safety, and acceptability, alongside the standard BTD provided with HAT RDT kits, in the hands of HWs performing HAT screening in northwestern Uganda, through observations, interviews, and focus group discussions (FGDs).

## METHODS

### The conical cup BTD.

**Figure 2.**
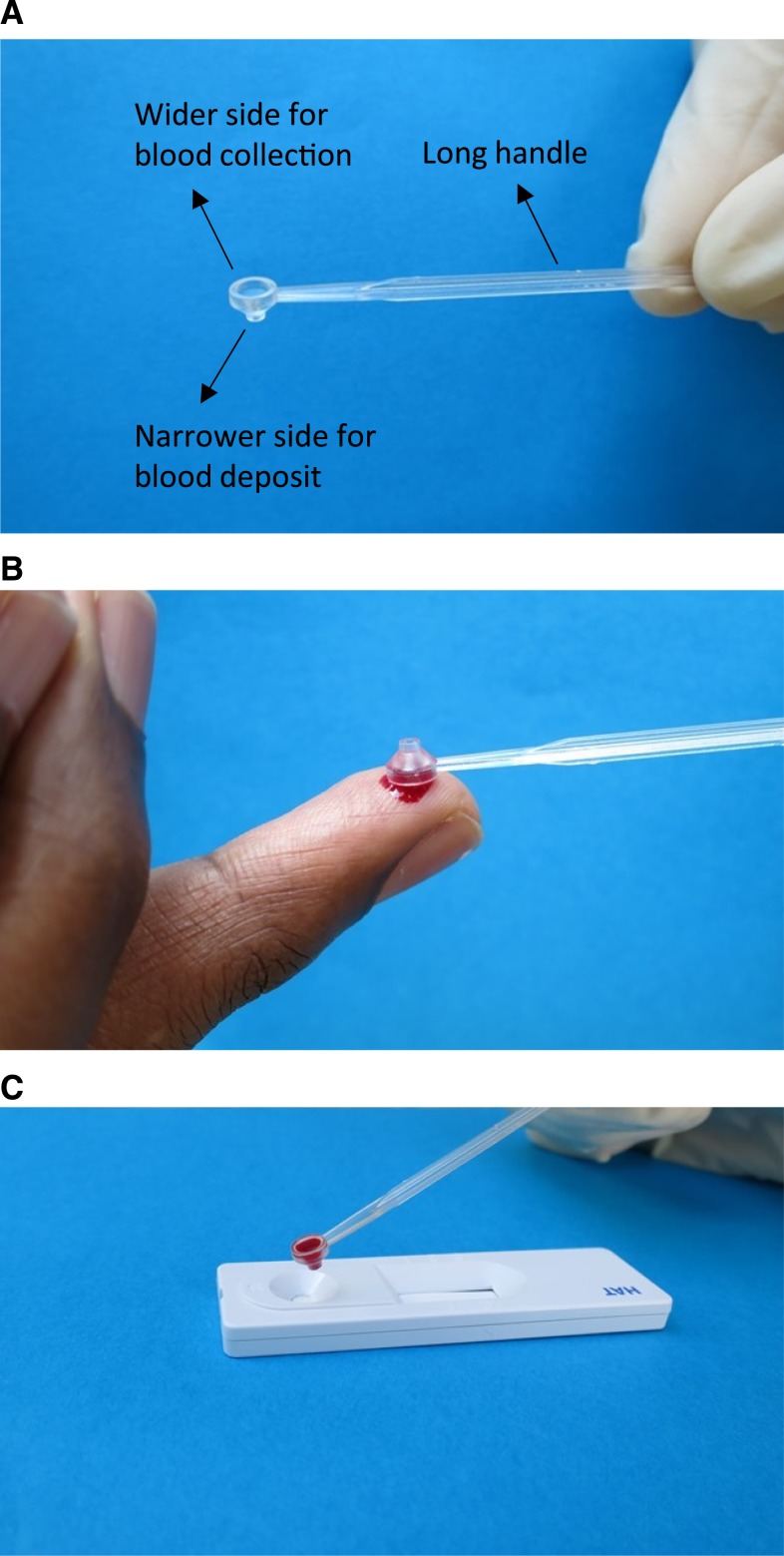
Photos of the conical cup blood transfer device, showing a general view (**A**), the blood collection step (**B**), and the deposit of blood on a rapid diagnostic test (**C**). This figure appears in color at www.ajtmh.org.

The conical cup BTD was developed by the FIND, Geneva, Switzerland, in collaboration with Injection 74, Alex, France. The conical cup BTD was initially designed to collect and transfer 20 μL of blood; however, the final manufactured device effectively transferred 23 μL (see following text). The device is made of styrene butadiene copolymers of K-Resin, KR03 grade, with a 6.9 cm long handle, and a neck of 8 mm. Figure 2A shows that it is open on both sides, with the wider opening used for blood collection (Figure 2B) and a narrower one for blood deposition (Figure 2C). The outer diameter of the wide side of the cup is 6 mm (inner diameter 4 mm), whereas the outer diameter of the narrow side of the cup is 2 mm (inner diameter 1 mm). The total height of the cup is 4 mm.

For the development of this device, at least three different prototypes were evaluated in an initial phase, including a conical cup, an inverted oval-shaped cup, and a round cup. The most promising, the “conical cup–type” design, was further explored by evaluating eight different designs of varying shapes and dimensions. Two of these eight designs were then selected for production and testing. A batch of 1,000 pieces was then manufactured with the final selected design, and the devices were then used for a precise volume determination study and in a field evaluation study in Uganda.

### Volume determination and accuracy of the conical cup BTD.

The volume determination study was conducted by experienced technicians at the National Tuberculosis Reference Laboratory, Kampala, Uganda, based on three different brands of HIV RDTs: the Determine HIV-1/2 Ag/Ab Combo test (Abbott Diagnostics, Santa Clara, CA), the HIV 1/2 STAT-PAK^™^ Assay (Chembio Diagnostic Systems, Inc., Medford, NY), and the Uni-Gold^™^ Recombigen^®^ HIV Rapid Test Kit (Trinity Biotech Plc, County Wicklow, Ireland). A single sample of venous blood from a volunteer blood donor, pre-screened for blood-borne infectious agents, was provided in an ethylenediaminetetraacetic acid (EDTA) tube. Using a calibrated micropipette, 20 μL aliquots of blood were spotted onto the blood sample well of 10 RDT cassettes from each of the abovementioned RDT brands. The weight of the individual RDT cassettes was determined both before and after having transferred the blood samples, using a calibrated precision balance, and the difference was recorded for each transfer. For evaluation of the conical cup BTD, small volumes (e.g., 50 μL) from the same blood sample were transferred to a gloved fingertip to simulate finger prick blood samples. Three different technicians used the conical cup BTDs to conduct 10 transfers of blood from simulated finger prick samples to 10 RDT cassettes from each of the three abovementioned HIV RDT brands, using a new conical cup BTD for each transfer. Individual RDT cassettes were similarly weighed before and after each blood transfer and weight differences were recorded. All weighing measurements were performed by a single dedicated technician.

### Field study setting and study sites.

The study was conducted from December 2013 to March 2014 as part of the project “Strategy for detection of *Trypanosoma brucei gambiense* HAT in northwestern Uganda: innovative strategy for *Tbg* HAT elimination” (the “HAT detection study”). This project implemented an enhanced passive surveillance strategy for HAT in northwestern Uganda using new RDTs in the public health system. To identify project sites to be upgraded to perform microscopy, all public and private not-for-profit health care facilities in the region were mapped using a hand-held global positioning system. The map generated was then used with other characterization data to identify strategically located health facilities. Health facilities were selected on the basis of 1) being accessible by road, 2) being well-equipped, and 3) having staff in the laboratories able to perform parasitological examination. All health facilities in the project area were equipped to screen for HAT using RDTs.

### Study population and sample size.

Health workers were invited to participate in the study if their health facility had been mapped and selected as a diagnostic center meeting the following selection criteria: established use of RDTs for HAT diagnosis, at least five HAT suspected patients seen per month, and availability of records or a logbook with data on RDT use, patient diagnoses, and treatments. Basic health care in the study areas is provided by nurses, clinical staff, and laboratory technicians. The term “HW” is used here to include both clinic staff and laboratory technicians as participants in the study. A sample size of approximately 50 HWs in the study region was targeted by inviting all those previously enrolled to provide HAT screening in the selected health facilities, and including those having provided informed and signed consent. Each participant was expected to perform 10 blood transfers using the conical cup BTD, giving a total sample size of 500 observations.

### Health workers prior experience with BTDs and training in use of the conical cup BTD.

In the framework of the “HAT detection study,” more than 200 health facilities were supplied with HAT RDTs (SD BIOLINE HAT; Abbott Diagnostics), and the HWs were trained in their use. The HWs using the HAT RDT were first trained on the squeezable pipette packaged with the test that, according to manufacturer’s instructions, measures approximately 20 μL of blood. The HWs enrolled for the BTD evaluation study were then invited to participate in a dedicated training workshop after completing a brief questionnaire to record their age, gender, level of qualification, and prior experience with BTDs. They were then trained in the use of the conical cup BTD, with a standardized half-day training package presented by members of the study team, including job aids specifically developed for this study (Supplemental File 1). Trainings were typically held for groups of 12–20 HWs at four health centers within the study area. After training, participants were invited to practice transferring blood with a maximum of 10 conical cup BTDs and 10 squeezable pipette BTDs. To this end, venous blood was collected in an EDTA tube from a volunteer donor having provided written informed consent, and drops of 50 μL of blood were placed on plain latex paper. Health workers then transferred blood to HAT RDT cassettes using both devices. Any questions and difficulties were discussed during and at the end of the training.

### Assessment of HWs’ perceptions and competency for use of RDTs and BTDs, at study start and at study end.

Immediately after the training, the HWs’ ability to correctly use the conical cup and the pipette BTDs was assessed by the staff from the study team. While observing the HWs during the testing of a HAT RDT using each of the two devices, their performance or correct completion of various critical steps was recorded using a standardized checklist (Supplemental File 2). Health workers had free access to the job aid, and any observed errors or difficulties of the HWs were not adjusted by the study staff to avoid biasing the assessment. Errors were explained to all trainees after completion of the assessment and the correct use of the BTDs emphasized once more. The HWs were also interviewed to evaluate their perceptions about the ease of use, the safety, and the suitability of the two different BTDs that they had used during the training. The members of the study team recorded their responses in a standardized questionnaire, using ordinal scales for most of the questions (e.g., ranging from “very easy” to “very difficult” for ease of use), whereas a few other questions allowed HWs to freely express their opinions (e.g., “what did you like/not like” about the BTD) (Supplemental File 3). The assessment of HWs correct use of the conical cup and pipette BTDs was repeated again at the end of the study, 3 months later, using the same standardized checklist (Supplemental File 2).

### Routine use of BTDs and recording of ease-of-use data by HWs.

After the initial assessment, 10–12 conical cup BTDs were given to each participating HW for use in their routine patient diagnostic setting. Standardized forms for recording the outcome of BTD use were provided, and HWs were given specific guidance on when and how frequently to use the new conical cup BTD, in the context of the on-going “HAT detection study.” For the purpose of this study, HWs performed two HAT RDTs on finger prick blood from each patient with symptoms suggestive of HAT, by transferring blood with the standard squeezable plastic pipette BTD on one of the RDTs and using the conical cup BTD for the blood transfer on the other RDT, from the same finger prick. This “double transfer” was only performed on the first 10 patients recruited for the “HAT detection study” to obtain a total of 10 blood transfers using each device. To avoid any bias because of the order of using one or the other of the two devices, it was decided to use the squeezable plastic pipette BTD first on all Tuesdays and Thursdays, whereas the conical cup device was used first on Mondays, Wednesdays, and Fridays. After each transfer, the HWs completed the record form as instructed (Supplemental File 4).

### Qualitative data collection.

At the end of the 3-month study period, FGDs and individual semi-structured interviews were held to gather qualitative information on HWs’ experiences with and perceptions of the two BTDs. Participating HWs were asked to report their perceptions about the two BTDs using ordinal scales and to suggest improvements, using the same questionnaire that was used for assessing their opinions immediately after the training workshop at the start of the study (Supplemental File 3). Four FGDs with about eight participants each were conducted following topic guides specifically developed for this purpose. Focus group discussions were conducted in English, which is the official training and communication language for all trained and qualified HWs in Uganda. All the FGDs and interview discussions were also recorded on audio files to facilitate data recording, cleaning, and analysis.

### Data management and statistical analysis.

For the volume determination study, the weight differences between RDT cassettes before and after each blood transfer were recorded on a dedicated Microsoft Excel spreadsheet, and mean values and standard deviations (SDs) were calculated for each set of data (i.e., transfers performed by each technician, with a micropipette or conical cup BTDs, with each brand of HIV RDTs) and for all values overall. The final volume transferred with the conical cup BTD was calculated using the density of blood of 1,060 kg/m^3^, based on the mean values obtained from all 30 transfers on the three RDT brands (i.e., a total of 90 transfers).

For the field study, data collection was conducted by a group of four study team members who were trained for 3 days before the field work to ensure that the steps for RDT and BTD use were precisely observed, interview questions were appropriately asked and observations or responses were consistently recorded. All data collection forms were reviewed daily by the study coordinators for completeness and accuracy. For qualitative data such as opinions expressed during the FGDs and interviews, the audio files were transcribed into text files and content analysis was performed manually, whereas NVIVO QDA Mac Beta 2014 software (QSR International Pty Ltd, Doncaster, Victoria, Australia) was used to group key findings into themes and subthemes. Themes that emerged from the data were categorized around a general understanding of ease of use and accurate performance.

Quantitative data collected on standardized forms with ordinal scales or yes/no responses were double-entered using EpiData (EpiData Association, Odense, Denmark) and analysis was performed using SPSS version 22 software (IBM Corporation, Armonk, NY). The study outcomes were presented as proportions and frequencies, and comparisons or changes in performance were assessed using Pearson’s χ^2^ or Fisher’s exact test, as indicated.

### Ethics considerations.

The study protocol was approved by the Vector Control Division Ethical Committee of the Uganda Ministry of Health and Uganda National Council for Science & Technology. All participating HWs and all enrolled patients provided written informed consent to participate in the study. Written consent was also provided by the volunteers donating blood for the volume accuracy study and the training workshops of the field study. District officials and health unit directors were informed about the study and were given the opportunity to visit the study sites. All participants were identified by coded study numbers, not names. The study team members were the only ones with access to the collected information.

## RESULTS

### Volume determination and accuracy of the conical cup BTD.

The conical cup BTD transferred an average blood volume of 22.76 μL (SD 3.31 μL) (Table 1). There were no significant differences between volumes transferred on the three types of HIV RDTs used for this study, nor between the three technicians’ results, suggesting robustness of the volume transferred with this device.

**Table 1:** Volume determination and accuracy of the conical cup BTD

	RDT kit 1		RDT kit 2		RDT kit 3		All RDTs	
	Mean	SD	Mean	SD	Mean	SD	Mean	SD
Technician 1 (g)	0.02508	0.00285	0.02405	0.00358	0.02747	0.00196	0.02553	0.00313
Technician 2 (g)	0.02667	0.00418	0.02234	0.00157	0.02396	0.00170	0.02432	0.00322
Technician 3 (g)	0.02106	0.00138	0.02555	0.00376	0.02093	0.00308	0.02251	0.00356
All technicians (g)	0.02427	0.00378	0.02398	0.00330	0.02412	0.00353	0.02412	0.00350
Blood volume (μL)	22.90	3.57	22.62	3.12	22.75	3.33	22.76	3.31

BTD = blood transfer device; g = grams; HIV = human immunodeficiency virus; RDT = rapid diagnostic test; SD = standard deviation; μL = microliter. Weight differences (in grams, g) between HIV RDT cassettes from three different brands before and after blood deposit using the conical cup BTD, and conversion into blood volume (in microliters, μL) according to blood density of 1,060 kg/m^3^ (blood volume = weight/1,060 × 1,000,000). RDT kit 1 = Determine HIV-1/2 Ag/Ab Combo test (Alere); RDT kit 2 = HIV 1/2 STAT-PAK^™^ Assay (Chembio Diagnostic Systems, Inc.); RDT kit 3 = Uni-Gold^™^ Recombigen^®^ HIV Rapid Test Kit (Trinity Biotech Plc).

### Participant HWs.

A total of 48 participants were enrolled in the study, based in 34 health facilities in the West Nile region, in the regions served by Yumbe Hospital, Omugo HC IV, Rhino Camp HC IV and Arua regional referral hospitals. Most participants were male (36/48, 75%) and about 76% (31/41) were laboratory assistants or technicians (Table 2). All participants had received prior training in the use of malaria RDTs, and a majority (41/42, 98%) had used them in routine patient care, over a median period of 6.5 months. Nearly all (46/48, 96%) had used various BTDs previously, with 98% having used the plastic pipette, 50% the capillary tube, and only 4% the inverted cup.

**Table 2:** Characteristics of participating health workers

Feature	Number (%) unless otherwise indicated
Number of participants	48
Age in years: median, minimum-maximum	27.5, 20–56
Female	12 (25%)
Male	36 (75%)
Professional category (*N* = 41)	
Laboratory technician/technologist	7 (17%)
Laboratory assistant	24 (59%)
Enrolled/registered nurse	10 (24%)
Highest educational level achieved (*N* = 37)	
O-level/certificate	29 (78%)
Diploma/university	8 (22%)
Formally trained in RDT use (*N* = 48)	48 (100%)
If trained, approximate number of months ago: median, minimum-maximum	2, 1–84
Has used any RDTs in routine patient care (*N* = 42)	41 (98%)
If RDTs used, approximate number of months used: median, minimum-maximum	6.5, 1–74
Prior use of any blood transfer device before evaluation date (*N* = 46)	
Loop	17 (37%)
Capillary	23 (50%)
Plastic pipette	45 (98%)
Straw	9 (20%)
Inverted cup	2 (4%)

RDT = rapid diagnostic test.

### Self-rated ease of use and safety of the two BTDs.

**Figure 3.**
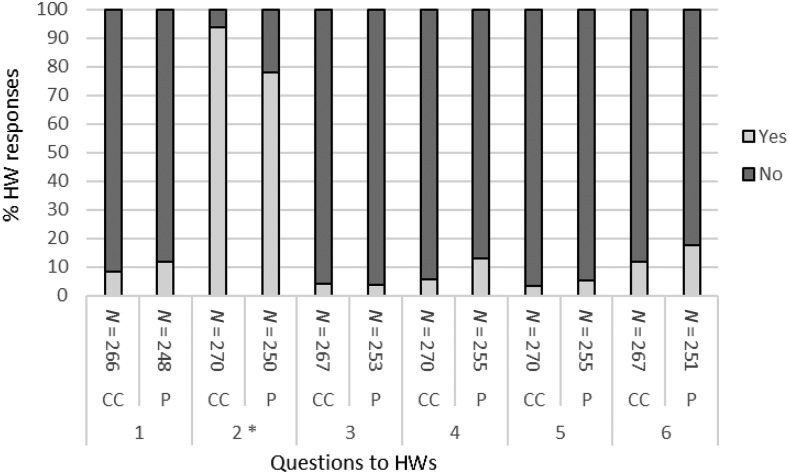
Health worker (HW) self-assessment responses comparing the conical cup and the pipette blood transfer devices (BTDs) immediately after use. Questions to 48 HWs conducting a maximum of 10 blood transfers each on human African trypanosomiasis rapid diagnostic tests (RDTs) with the conical cup and the pipette BTDs for the diagnosis of patients were as follows. 1) Did you have to make more than one attempt to collect the desired amount of blood? 2) Was the conical cup fully filled with blood, or the pipette filled to the marked line? 3) Was blood released unintentionally from the device at any time before reaching the RDT? 4) Did you have to make more than one attempt to deposit all the blood in the RDT well? 5) Did blood touch your gloves, skin, clothing, or any other surface at any time? 6) Was there any blood remaining in the transfer device after deposit in the RDT well? CC = conical cup BTD; P = pipette BTD. *Signifies comparison between the pipette and the conical cup BTDs being filled adequately with blood; OR = 3.42 (1.89–6.21), *P* < 0.001 (McNemar’s test).

Health workers’ self-recorded observations suggest that few issues were encountered regarding blood safety, with close to 95% of transfers reported without any unintentional blood release or blood exposure to the HW, with very similar results for both BTDs (Figure 3). Recorded success rates were also good (> 90%) for the collection and deposit of the blood in only one attempt, again with similar results obtained for both BTDs. Transfers without any visible blood residue remaining in the BTD after blood deposit were reported to be less frequent, but still greater than 80%, with no difference between the BTDs. The correct filling of the BTD, however, was significantly easier with the conical cup as compared with the pipette (90% versus 78%, odds ratio [OR] = 3.42 (1.89–6.21), *P* < 0.001).

### Observed ease of use and safety of the two BTDs.

**Figure 4.**
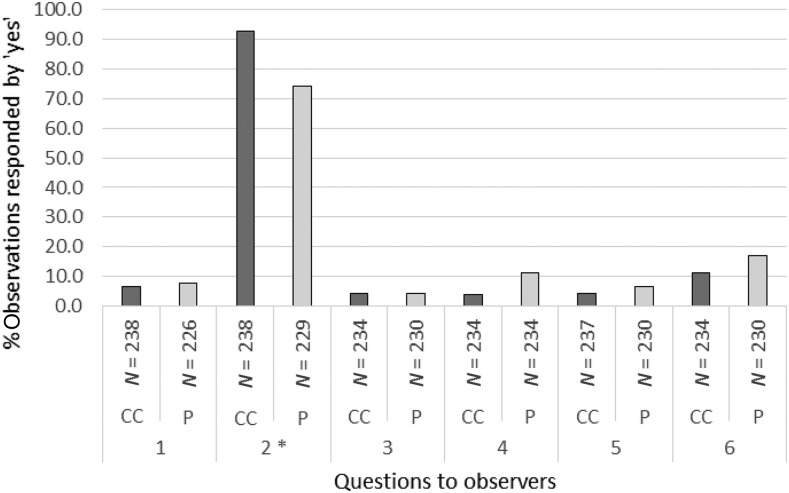
Ease of use and safety of the pipette and the conical cup blood transfer devices (BTDs) in the hands of health workers (HWs), assessed by observers at study end. Questions to study staff observing HWs conducting a maximum of five blood transfers on human African trypanosomiasis rapid diagnostic tests with the conical cup and the pipette BTDs during a workshop at the study end were as follows. 1) Did the HW make more than one attempt to collect blood? 2) Was the cup fully filled with blood/the pipette filled up to the mark? 3) Was blood released unintentionally? 4) Did the HW make more than one attempt to deposit blood? 5) Did blood touch HW’s gloves, skin, clothing, or surfaces? 6) Did blood remain in the conical cup/in the pipette BTD after deposit? CC = conical cup BTD; P = pipette BTD. *Signifies comparison between the pipette and the conical cup BTDs being filled adequately with blood; OR = 3.42 (1.89–6.21), *P* < 0.001 (McNemar’s test).

At the end of the study, the ease of use and safety of the two BTDs were evaluated by members of the study team observing the HWs performing blood transfers with the two devices. The observers completed questionnaires with the same type of questions as the ones which HWs answered during their self-assessment. The results, shown in Figure 4, mirror the ones obtained in the HW self-rating, i.e., the conical cup BTD performed better in terms of adequate blood filling (92.4% versus 74.2%, OR = 3.42 (1.89–6.21), *P* < 0.001). It was nevertheless reported that some HWs did multiple “touch-downs” (i.e., lifting the conical cup BTD and touching the RDT pad again) to successfully deposit all blood in the RDT sample well.

### Health worker perception about ease of use and safety of the two BTDs.

**Figure 5.**
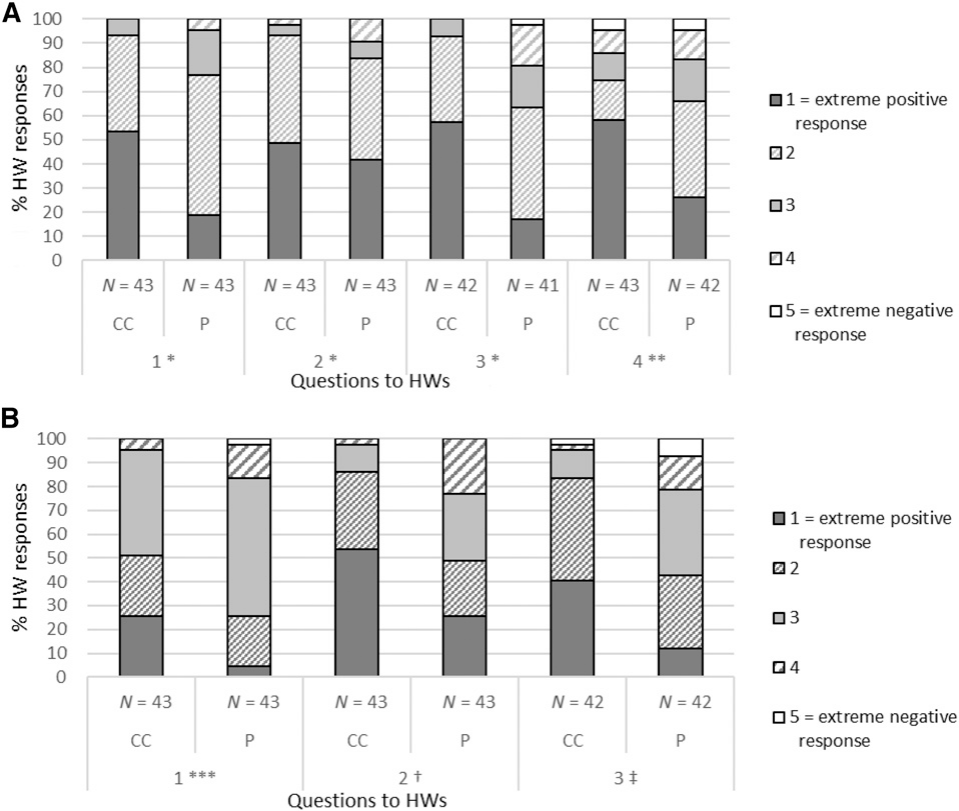
Opinions of health workers (HWs) about the ease of use, safety, and appropriateness of the pipette and the conical cup blood transfer devices (BTDs), at study end. Questions asked to HWs during a workshop at the study end were as follows. (**A**) 1) What was the ease of collection of blood from the patient finger prick? 2) What was the ease of releasing (depositing) blood into the rapid diagnostic test well? 3) What was the difficulty in handling? 4) What was the risk of blood exposure? (**B**): 1) What was the effect on the speed of work? 2) What was the effect on confidence in front of the patient? 3) Is the BDT appropriate for HWs to use in patient care? *1 = very easy; 2 = easy; 3 = not so difficult; 4 = difficult; 5 = very difficult. **1 = no risk; 2 = very little risk; 3 = little risk; 4 = quite some risk; 5 = great risk. ***1 = extremely fast; 2 = very fast; 3 = it was at the normal pace; 4 = slow; 5 = very slow. †1 = very confident; 2 = more confident; 3 = it did not affect my confidence; 4 = less confident; 5 = not confident at all. ‡1 = excellent; 2 = very appropriate; 3 = appropriate; 4 = little appropriate; 5 = not appropriate.

Health worker perception, as recorded by study staff in individual interviews at the end of the study, is shown in Figure 5. For the conical cup BTD, 93% of the HWs reported that they found the device easy or very easy to use for blood collection, blood deposit, and for handling in general, against 77%, 84%, and 66% of HWs for the pipette BTD, respectively (Figure 5A). Larger proportions of HWs also reported 1) feeling more confident in front of the patient with the conical cup BTD, as compared with the pipette BTD (86% versus 49%), 2) that the device speeds up their work (51% versus 26%), and 3) that it is appropriate for use in routine patient care (Figure 5B). When asked which device would be best suited for every day work, 79% of HWs reported that they preferred the conical cup BTD. Only 9% reported preferring the pipette BTD, whereas 11% said they considered both devices well suited. The most common reason noted by HWs for preferring the conical cup device was that there is no need to “actively” control the blood volume.

### Health worker opinions about the BTDs, expressed in FGDs.

The qualitative data collection was used to reinforce the understanding of observations recorded during the use of BTDs and during the interviews. Box 1 (Supplemental File 5) shows a list of quotes which reflect the most common themes expressed by HWs during the FGDs, as pertaining to the conical cup and pipette BTDs. Various challenges using the BTDs were described, such as the difficulty using the pipette to collect blood, to control the blood volume by aspiration, and the risk of air bubbles and blood spillage (“the problem there is controlling that pressure is not easy”; “collects unnecessary bubbles especially when you apply a lot of pressure,” and “when dispensing there is a tendency of blood dropping before it reaches the right spot”). Concerns mentioned for the conical cup BTD included the blood deposit, especially if the cup was not entirely filled (“if you don’t fill it well, there will be a problem touching the spot and blood will not come out”). Positive opinions about the pipette BTD included that it was easier to use because of familiarity (“my work has been easier with the pipette, maybe because I have used it for long”). The most common advantage mentioned for the conical cup device was the automatic volume control without any pressure or other specific manipulation (“you don’t need pressure, you just put [place the device on the blood drop] and it automatically fills”; “it is just a matter of touching the blood and then it comes up by itself and then just transferring it on the pad”).

## DISCUSSION

The deployment of appropriate BTDs with RDTs is expected to enhance the overall accuracy and reliability of POC diagnosis, and minimize risk of blood exposure for HWs. This study looked not only at the accuracy of the blood volume transferred by the new conical cup BTD but also at three parameters of concern in the transfer of blood to the RDTs—ease-of-use, safety, and acceptability to HWs. The volume determination study provides a good estimate of the typical blood volume transferred with this device in good laboratory conditions, and when used with different brands of HIV RDTs. Volume variation in the hands of a limited number of HWs was small, and larger studies with a higher number of technicians could be useful to confirm the volume robustness of this device. However, important volume variations would not be expected because the volume is essentially determined by the device’s design, rather than by HW skill.

The field evaluation study was purposely conducted in a POC setting where ease-of-use tests and devices are most needed and can have the most impact. The characteristics of enrolled study participants fit well with the target population for use of the new BTD, i.e., consisting mostly of laboratory technicians and assistants but also nurses, with prior experience using RDTs in routine patient care, and capacity to adequately perform the most critical steps of testing with RDTs.

The quantitative data of this study demonstrated that the new conical cup BTD offered various advantages over the plastic pipette, such as 1) improved HW performance in adequately collecting the required blood volume and releasing the full blood volume onto the RDT pad and 2) improved ease of use, particularly for the blood collection step. The qualitative data collected during individual interviews and FGDs at the end of the study reinforce the views that the new conical cup BTD was highly acceptable, convenient, and perceived as being easier for routine use with RDTs compared with the pipette. Health workers also reported that work was faster while using the conical cup device, that they felt very confident, and that they found the conical cup BTD to be appropriate for use in routine patient healthcare. Most HWs (nearly 80%) stated that the conical cup BTD would be their preferred choice for routine patient care.

Explanations from the HWs indicated that the conical cup BTD’s major advantage is that it does not require any active volume adjustments during sample collection, because this happens by capillary action, and the sample is then released through simple contact with the RDT filter pad. By contrast, the pipette BTD necessitates aspiration of the blood with careful control to collect it up to the mark, then an application of extra pressure to expel the blood sample is required for depositing it in the RDT.

The observations made, and comments collected, in this study about the conical cup BTD are useful for training, a critical aspect for appropriate RDT implementation.^8,12^ For example, training tools should highlight that the blood deposit should be performed by a light touch on the filter pad with the BTD followed by a slow lifting of the device, easily allowing the entire blood volume to be absorbed by the RDT filter pad.

This study was conducted in the context of day-to-day clinical care with HWs serving actual patients and having to deliver a proper diagnosis. In POC settings, routine clinical work is often not performed in controlled environments, and conditions may not be ideal to successfully carry out a clean blood transfer, as with a fidgeting crying child. For example, in this study, the shape of the pipette may have affected its use. When patients thought that the pipette was another “pricker” (lancet, thus fear of pain), it further compounded the difficulty in blood collection. For HWs, aspects like the ability to work rapidly, feeling confident in front of the patient, and not facing any difficulty using the device or blood exposure while they are performing the diagnostic test, are critical and therefore more easily measured in such a setting.

One limitation of this study is that the conical cup BTD was compared with only one other device, the plastic pipette. The difference between these two devices (the conical cup BTD allows for collecting and depositing blood without any “active” volume control, whereas the pipette requires specific manipulation to control the volume) may explain the marked preference for the conical cup BTD over the pipette. To the authors’ knowledge, there exist no other devices which allow for automatic collection of 20 μL blood volume to which the conical cup BTD could have been compared. Another limitation is that this study has focused on a sample of 50 HWs in only one country and study area; obviously, it would be useful to repeat such a study with larger sample sizes and in various geographical settings, e.g., other African countries, or on other continents such as Asia and South America. Finally, a third limitation is that the current conical cup device design is for 23 μL instead of 20 μL which is the standard target volume for various RDTs. Funding limitation did not allow further refining the BTD design for the exact volume of 20 μL. To reach a precise 20 μL volume, extensive testing of a variety of prototypes with human capillary blood is required, with a fine adjustment of the cup’s dimensions and an adequate choice of the plastic material. Unfortunately, there are no specific studies published on what would be an acceptable deviation from the target volume. Foundation for Innovative New Diagnostics, in the frame of a project on malaria RDT implementation in the private sector, had informal communications with manufacturers of malaria RDTs, and acceptable deviations of 5–10% were cited by some (i.e., 0.25–0.5 μL for a target volume of 5 μL). Determination of the acceptable volume variation for RDTs using 20 μL volumes would require dedicated studies or formal statements made by the RDT manufacturers themselves. In summary, the use of the 23 μL conical cup device for RDTs designed for 20 μL blood volume would obviously require some prior validation that the quality of the tests are not affected by the 3 μL excess volume or alternatively require some adjustments of the design to decrease the volume to 20 μL of blood.

Previous studies comparing BTDs for malaria RDTs with a transfer of 5 μL of blood have assessed their ease of use and accuracy.^16,17^ The results demonstrated the success of the 5 μL inverted cup BTD with regard to its accuracy and ease of use, especially for the blood collection step.^17^ The 23 μL conical cup BTD features a new design for a larger volume of blood which is similarly accurate, perceived as easier to use, and results in better performance for the blood collection and blood deposit steps in the hands of HWs in lower level health facilities, as compared with the pipette BTD. Larger studies, including comparisons with other 20 μL BTD devices, would further strengthen these findings. However, the present study already provides good indications that the innovative design of the conical cup BTD is well adapted for easy and safe use with POC diagnostics requiring such blood volumes.

## Supplementary Material

Supplemental files
