# Supplementary material for: Accuracy, Ease of Use, Safety, and Acceptability of a 23-μL Conical Cup Blood Transfer Device for Use with Rapid Diagnostic Tests
Source: Am J Trop Med Hyg. 2018 Jul 16;99(3):797–804. doi: 10.4269/ajtmh.17-0716 (PMC6169173; doi:10.4269/ajtmh.17-0716)
Supplement: Supplementary file 1 [file tpmd170716.SD1.pdf]

Study comparing blood transfer devices

Done using the rapid test for Human African Trypanosomiasis (HAT)

Modified for training and use of the ***SD BIOLINE HAT*** *test using plastic pipette and conical cup*

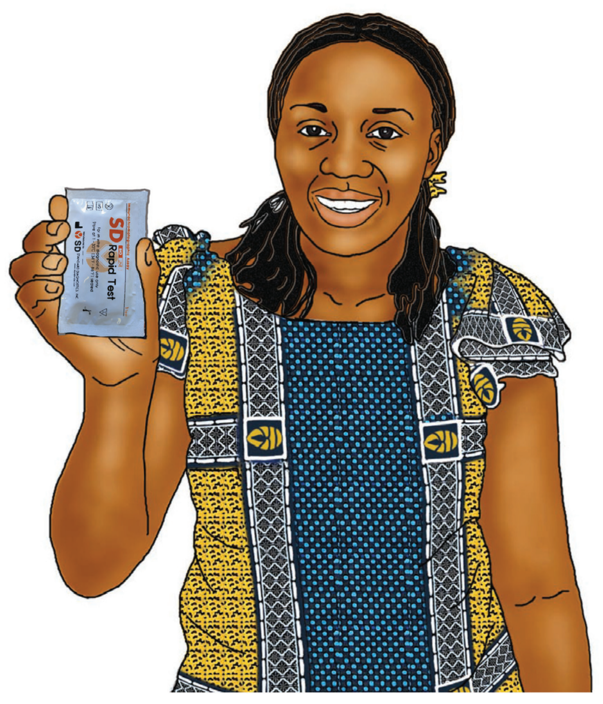

Take:

a. **NEW unopened** test packets (2)
b. **NEW** plastic pipette
c. **NEW** conical cup
d. **NEW unopened** alcohol swab
e. **NEW sterile** lancet
f. **NEW** pair of disposable gloves
g. Assay diluent
h. Timer
i. Sharps box
j. Pencil or pen

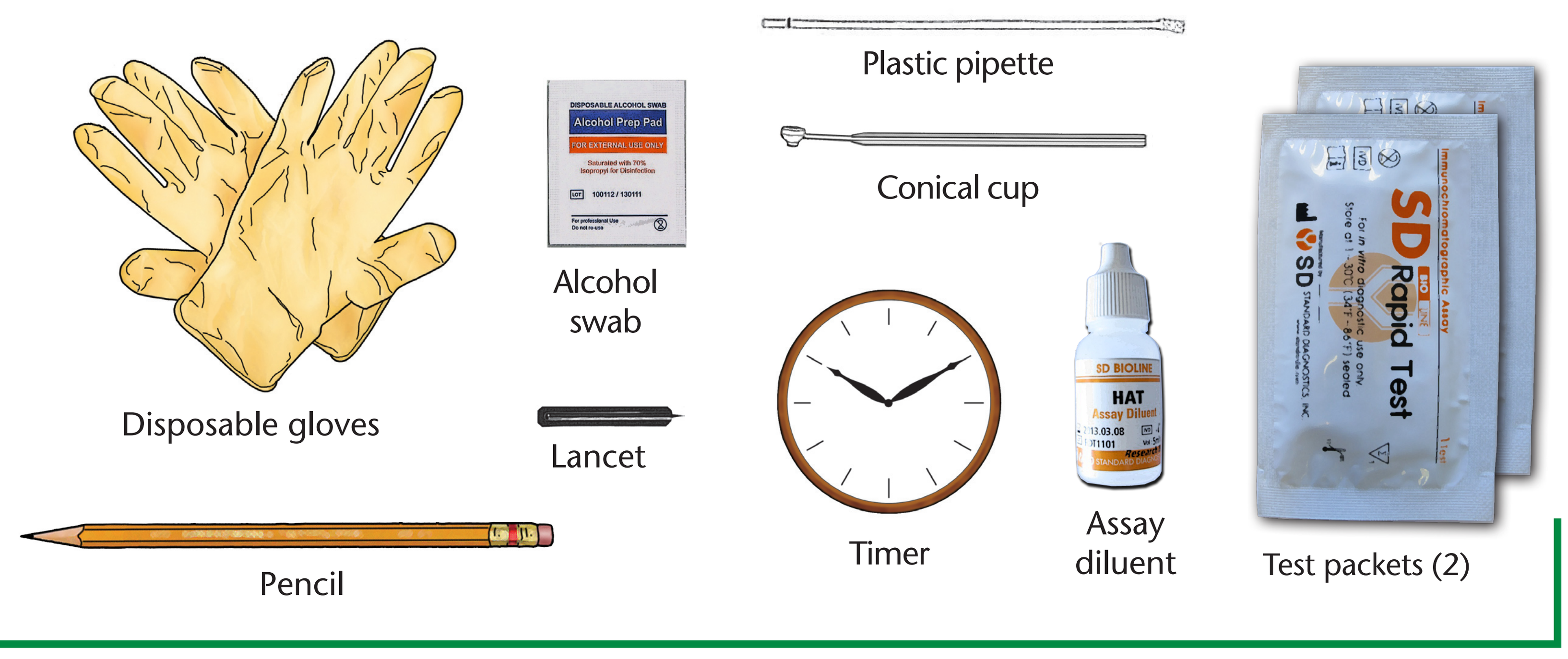

READ THESE INSTRUCTIONS CAREFULLY BEFORE YOU BEGIN.

1. Check the expiry date on each of the test packets.

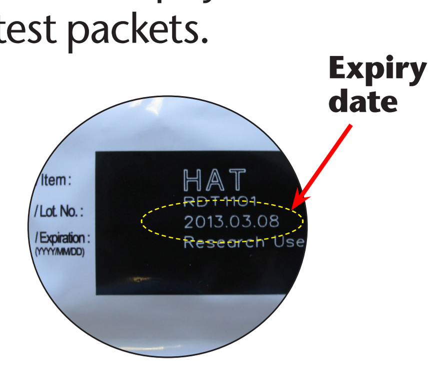

2. Put on the gloves. Use new gloves for each patient.

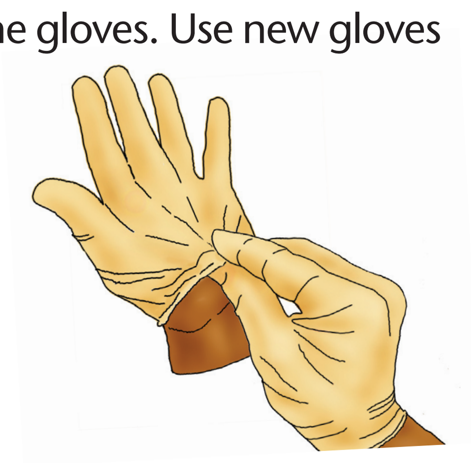

3. Open each of the 2 packets and remove:

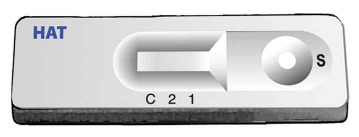

a. Test cassette

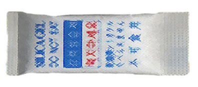

b. Desiccant

4. Label the tests 'HAT' and 'CC' and write the patient code on each.

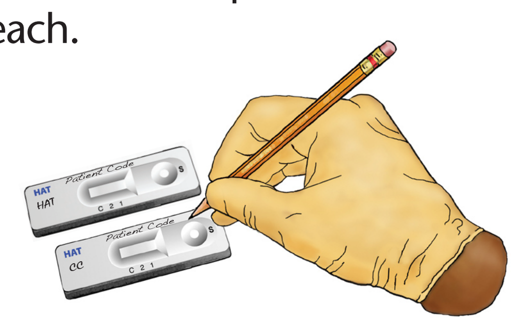

5. Place the two test cassettes on a flat surface.

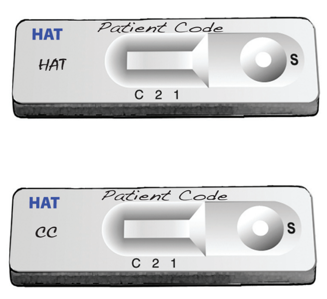

6. Open the alcohol swab. Grasp the 4th finger on the patient's left hand. Clean the finger with the alcohol swab. Allow the finger to dry before pricking.

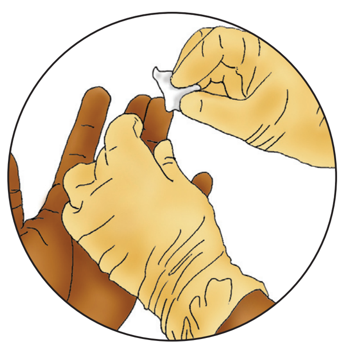

7. Open the lancet. Prick the patient's finger to get a drop of blood. Do not allow the tip of the lancet to touch anything before pricking the patient's finger.

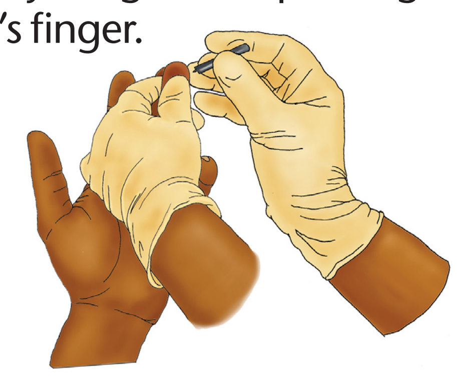

8. Discard the lancet in a sharps box immediately after pricking finger. **Do not set the lancet down before discarding it.**

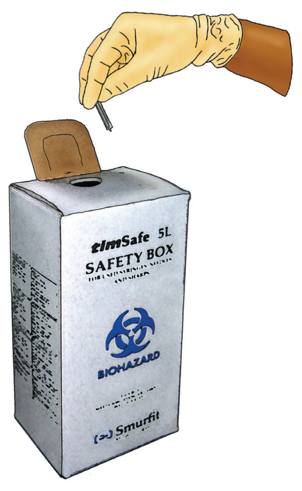

Comparative use of plastic pipette and conical cup as blood transfer device

9. Collect blood sample.

a. USING THE PIPETTE: Gently squeeze and touch the tip of the pipette to the drop of blood. Gently release the pressure to draw blood up to the black line on the pipette.

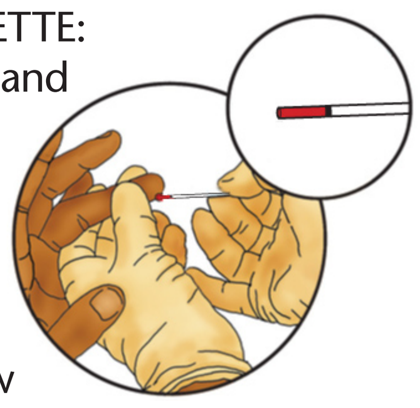

b. USING THE CONICAL CUP: Touch the drop of blood with the wider side of the cup.

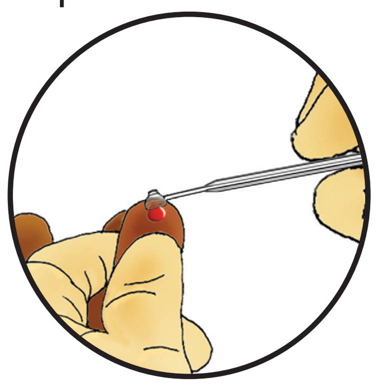

10. Transfer blood sample to cassette.

a. USING THE PIPETTE: Touch the tip of the pipette to the sample hole marked 'S' of the cassette labelled 'HAT'. Squeeze gently to transfer the blood.

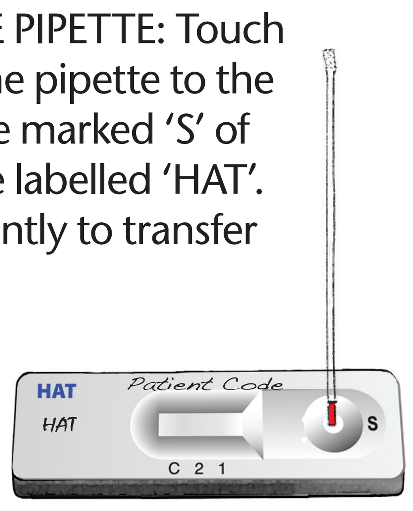

b. USING THE CONICAL CUP: Ensure the cup is completely full before beginning transfer. Deposit the blood into the sample well of the cassette labelled 'CC' directly using the narrower side of the conical cup.

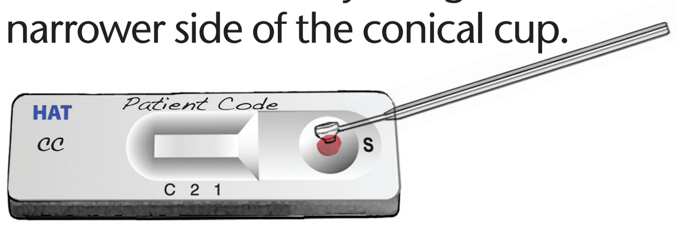

11. Discard the blood transfer device in the sharps box.

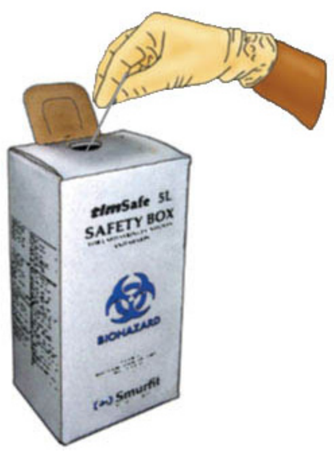

12. Put four (4) drops of assay diluent into the sample hole. Record the time after doing so.

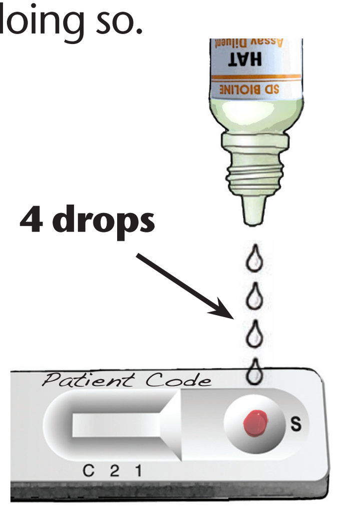

13. Wait 15 minutes after adding diluent.

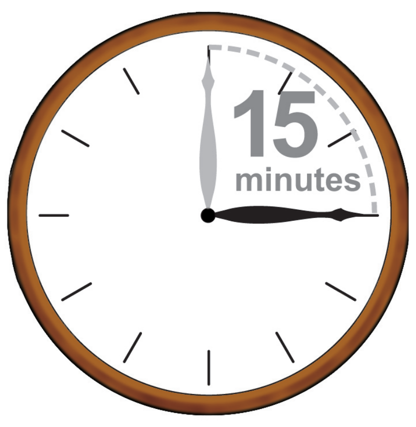

14. Read test result. Do not read sooner than 15 minutes after adding diluent. You may get a FALSE result.

NOTES:

Mon., Wed. & Fri. - Use the concical cup first, then the pipette.

Tue. & Thur. - Use the pipette first, then the conical cup.

Results of 'HAT' tests are to be used in patient care; results of 'CC' tests are for evaluation of conical cup.

15. How to read the test results:

POSITIVE

A line in **"C" AND** line in **"1"** and/or **"2"** means the patient is SUSPECT for HAT (presence of antibodies directed against HAT antigens). The test is **POSITIVE** even if the lines in **"1"** and/or **"2"** are faint.

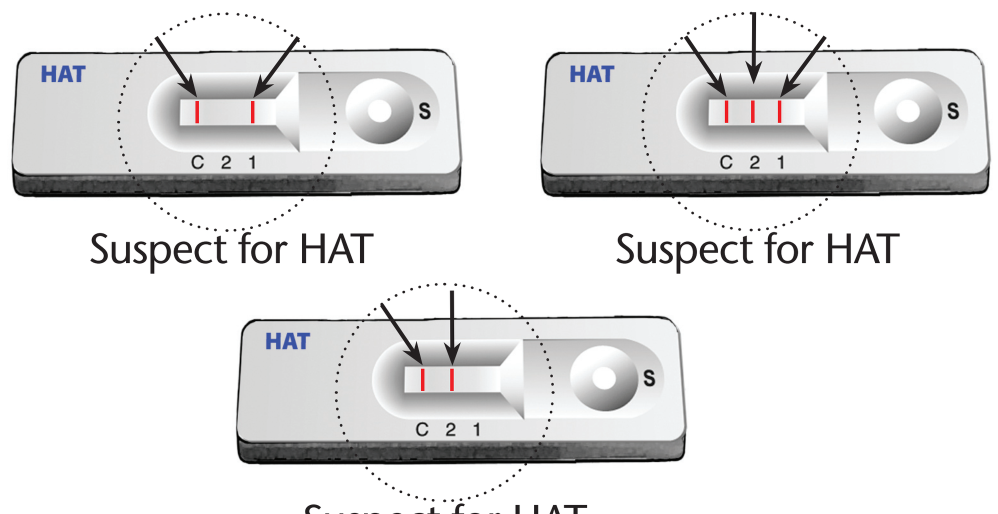

Suspect for HAT

Suspect for HAT

Suspect for HAT

NEGATIVE

A line in **"C"** and **NO LINE** in either **"1"** or **"2"** means the patient **DOES NOT** have HAT.

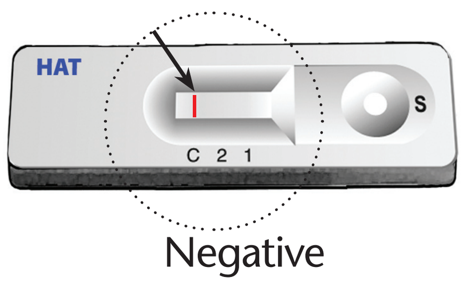

Negative

INVALID RESULT

NO LINE in **"C"** and a line or no line in **"1"** and/or **"2"** means the test is **INVALID**.

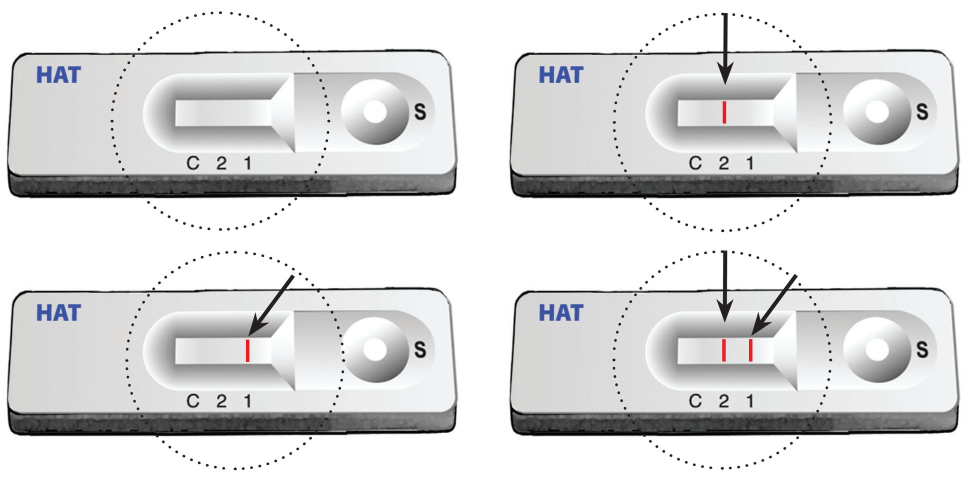

If no line appears in "C," repeat the test using a **NEW unopened** test packet and a **NEW unopened** lancet.

16. Dispose of gloves*, alcohol swab, desiccant sachet and packaging in a non-sharps waste container.

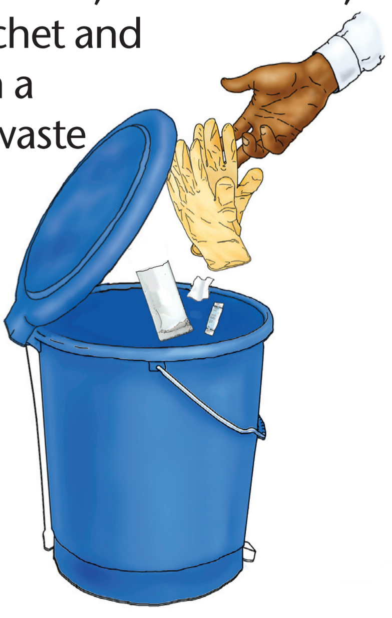

* In case of active screening, follow national guidelines.

17. Record the test result for RDT-HAT in the register then answer the questionnaire for RDT-CC. Discard the cassettes in the non-sharps waste container.

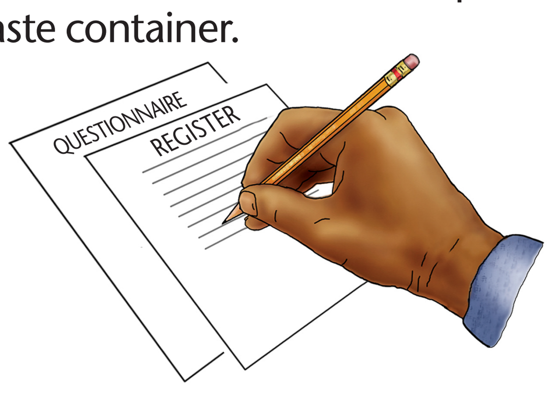

NOTE: Each test can be used **ONLY ONE TIME**. Do not try to use the test more than once.

FIND foundation for innovative new diagnostics

SD STANDARD DIAGNOSTICS, INC. www.standardia.com

Produced by the Foundation for Innovative New Diagnostics (FIND) from generic material developed jointly by the World Health Organization (WHO), United States Agency for International Development (USAID), University Research Co., LLC (URC), Special Programme for Research and Training in Tropical Diseases (TDR), Malaria Consortium and Zambia National Malaria Control Centre.

Prepared on 12 November 2013. Since manufacturers' instructions may have changed after this job aid was produced, all details should be cross-checked against manufacturer instructions in the product insert of the test in use.
