# Supplementary material for: Accuracy, Ease of Use, Safety, and Acceptability of a 23-μL Conical Cup Blood Transfer Device for Use with Rapid Diagnostic Tests
Source: Am J Trop Med Hyg. 2018 Jul 16;99(3):797–804. doi: 10.4269/ajtmh.17-0716 (PMC6169173; doi:10.4269/ajtmh.17-0716)
Supplement: Supplementary file 2 [file tpmd170716.SD2.doc]

**Supplementary file 2**

***‘Ease of use and safety of the conical cup blood transfer devices for use with rapid diagnostic tests (RDTs) for Human African Trypanosomiasis (HAT)’ study***

***Observer’s Form***

Kindly observe the following as regards the specific use of the Conical cup.

| Date/Day: |
| --- |
| Transfer with the conical cup was done: First Second |

| **Collection**  Did the health worker have to make more than one attempt to collect the desired amount of blood? | ** 🖵 Yes 🖵 No**  **If yes, kindly explain;** |
| --- | --- |
| **Amount of blood collected**  Was the cup of the conical cup fully filled with blood? | ** 🖵 Yes 🖵 No**  **If no, kindly explain;** |
| **Transfer**  Was blood released unintentionally from the conical cup at any time before reaching the RDT? | ** 🖵 Yes 🖵 No**  **If yes, kindly explain;** |
| **Deposit**  Did the health worker have to make more than one attempt to deposit all the blood in the RDT well? | ** 🖵 Yes 🖵 No**  **If yes, kindly explain;** |
| **Exposure**  Did blood touch the health worker’s gloves, skin, clothing or any other surface at any time? | ** 🖵 Yes 🖵 No**  **If yes, kindly explain;** |
| **Remaining blood**  Was there any blood remaining in the conical cup after deposit in the RDT well? | ** 🖵 Yes 🖵 No**  **If yes, kindly estimate;** |
