# Supplementary material for: Accuracy, Ease of Use, Safety, and Acceptability of a 23-μL Conical Cup Blood Transfer Device for Use with Rapid Diagnostic Tests
Source: Am J Trop Med Hyg. 2018 Jul 16;99(3):797–804. doi: 10.4269/ajtmh.17-0716 (PMC6169173; doi:10.4269/ajtmh.17-0716)
Supplement: Supplementary file 3 [file tpmd170716.SD3.doc]

**Supplementary file 3**

‘Ease of use and safety of the conical cup blood transfer devices for use with rapid diagnostic tests (RDTs) for Human African Trypanosomiasis (HAT)’ study

Participant ID No: Staff Initials:

Facility: Site:

Age: Sex:

Highest Qualification: Date (of qualification):

Designation: Date:

**Questionnaire:**

What do you think of the devices you evaluated today?

**Conical Cup**

1a) What was the ease of collection of blood from the patient finger prick?

1. Very Difficult
2. Difficult
3. Not so difficult
4. Easy
5. Very easy

2a) What was the ease of releasing (depositing) blood into the RDT well?

1. Very Difficult
2. Difficult
3. Not so difficult
4. Easy
5. Very easy

**Pipette**

1b) What was the ease of collection of blood from patient finger prick?

1. Very Difficult
2. Difficult
3. Not so difficult
4. Easy
5. Very easy

2b) What was the ease of releasing (depositing) blood into the RDT well?

1. Very Difficult
2. Difficult
3. Not so difficult
4. Easy
5. Very easy

**Conical Cup**

3a) What was the risk of blood exposure?

1. Great risk
2. Quite some risk
3. Little risk
4. Very little risk
5. No risk

4a) What was the effect on the speed of work? It made my work:

1. Very slow
2. Slow
3. It was at the normal pace
4. Very fast
5. Extremely fast

5a) What was the effect on my confidence in front of the patient? I was:

1. Not confident at all
2. Less confident
3. It did not affect my confidence
4. More confident
5. Very confident

**Pipette**

3b) What was the risk of blood exposure?

1. Great risk
2. Quite some risk
3. Little risk
4. Very little risk
5. No risk

4b) What was the effect on the speed of work? It made my work:

1. Very slow
2. Slow
3. It was at the normal pace
4. Very fast
5. Extremely fast

5b) What was the effect on my confidence in front of the patient? I was:

1. Not confident at all
2. Less confident
3. It did not affect my confidence
4. More confident
5. Very confident

**Conical Cup**

6a) What was the difficulty in handling?

1. Very Difficult
2. Difficult
3. Not so difficult
4. Easy
5. Very easy

7a) Kindly elaborate on your answer above: …………………………………………………………………………………………………………………………………………………………………………………………………………………………………………….…………………………………………………………

8a) Is the conical cup appropriate for health workers to use in patient care? It is:

1. Not appropriate
2. Little appropriate
3. Appropriate
4. Very Appropriate
5. Excellent

9a) Any other comment: ………………………………………………………………………………………………………………………………………………………………………………………………………………………………………………………………………………………………………..

**Pipette**

6b) What was the difficulty in handling?

1. Very Difficult
2. Difficult
3. Not so difficult
4. Easy
5. Very easy

7b) Kindly elaborate on your answer above: …………………………………………………………………………………………………………………………………………………………………………………………………………………………………………….…………………………………………………………

8a) Is the pipette appropriate for health workers to use in patient care? It is:

1. Not appropriate
2. Little appropriate
3. Appropriate
4. Very Appropriate
5. Excellent

9b) Any other comment: ……………………………………………………………………………………………………………………………………………………………………………………………………………………………………………………………………………………………………

10a) In your opinion, which of the two devices would be best suited for use with RDTs in everyday work?

………………………………………………………………………………………………………………………………………….………………………

10b) What is the main reason for your choice?

…………....................................................................................................................................................................................................................................................................................................................................

11a) Do you have any suggestions to improve any of the devices you evaluated today?

**** Yes **** No

11b) If Yes, kindly elaborate: ……………………………………………………………………………………………………………………………………………………………………………………………………………………………………………………………………………………………………………………………………

12a) Is there another device that we did not evaluate today that you particularly like?

**** Yes  **** No

12b) If Yes, kindly elaborate: ……………………………………………………………………………………………………………………………………………………………………………………………………………………………………………………………………………………………………………………………………

13a) If the conical cup was recommended for you to use to perform RDTs for your patients, would you use it?

**** Yes  **** No

13b) If No, kindly elaborate: ……………………………………………………………………………………………………………………………………………………………………………………………………………………………………………………………………………………………………………………………………

1. Can you teach a colleague how to use this blood transfer device?

**** Yes  **** No
