# Supplementary material for: Accuracy, Ease of Use, Safety, and Acceptability of a 23-μL Conical Cup Blood Transfer Device for Use with Rapid Diagnostic Tests
Source: Am J Trop Med Hyg. 2018 Jul 16;99(3):797–804. doi: 10.4269/ajtmh.17-0716 (PMC6169173; doi:10.4269/ajtmh.17-0716)
Supplement: Supplementary file 4 [file tpmd170716.SD4.doc]

**Supplementary file 4**

‘Ease of use and safety of the conical cup blood transfer devices for use with rapid diagnostic tests (RDTs) for Human African Trypanosomiasis (HAT)’ study.

To be completed immediately after transferring the blood and recording results in the register

| Participant ID No; | Date: | Week-day: |
| --- | --- | --- |
| **CC-RDT Result***: | 1^st^ Transfer done with:   Conical Cup  Pipette | 2^nd^ Transfer done with:   Conical Cup  Pipette |
| **Collection**  Did you have to make more than one attempt to collect the desired amount of blood? |  Yes  No  If yes, kindly explain; |  Yes  No  If yes, kindly explain; |
| **Amount of blood collected**  Was the cup of the device fully filled with blood? |  Yes  No  If no, kindly explain; |  Yes  No  If no, kindly explain; |
| **Transfer**  Was blood released unintentionally from the device at any time before reaching the RDT? |  Yes  No  If yes, kindly explain; |  Yes  No  If yes, kindly explain; |
| **Deposit**  Did you have to make more than one attempt to deposit all the blood in the RDT well? |  Yes  No  If yes, kindly explain; |  Yes  No  If yes, kindly explain; |
| **Exposure**  Did blood touch your gloves, skin, clothing or any other surface at any time? |  Yes  No  If yes, kindly explain; |  Yes  No  If yes, kindly explain; |
| **Remaining blood**  Was there any blood remaining in the transfer device cup after deposit in the RDT well? |  Yes  No  If yes, kindly estimate; |  Yes  No  If yes, kindly estimate; |

*** RDT performed with the conical cup device**
