# Supplementary material for: Accuracy, Ease of Use, Safety, and Acceptability of a 23-μL Conical Cup Blood Transfer Device for Use with Rapid Diagnostic Tests
Source: Am J Trop Med Hyg. 2018 Jul 16;99(3):797–804. doi: 10.4269/ajtmh.17-0716 (PMC6169173; doi:10.4269/ajtmh.17-0716)
Supplement: Supplementary file 5 [file tpmd170716.SD5.doc]

**Box 1:** **Quotes representing common themes expressed by health workers during focus group discussions**

**Quote 1 A1**: My personal experience with plastic pipette is when you make a small prick and blood is little, it is hard to fill but if the drop is sufficient it fills easily.

**Quote 2 A2:** My personal experience with the pipette is that first of all it needs pressure to be applied while obtaining the sample and if you are someone who is not confident enough measuring the exact amount will be a problem and also when releasing the blood into that spot you will find that some of the blood has splashed out.

**Quote 3: P1**: With the pipette, you need to be confident so if you are shaking, a drop of blood can easily splash on you

**Quote 4: G1:** The pipette when you get blood releasing it takes time, it might remain there, that is my experience with it is difficult to release blood.

**Quote 5: A2**: My experience tells me that the conical cup is more convenient, looking at the challenges of the capillary tube and pipette but with the conical cup; you don’t need pressure, you just put [place the device on the blood drop] and it automatically fills. There is nothing like spillage due to pressure reasons therefore collection of blood is very simple.

**Quote 6: T1:** With conical cup, there is no bubbling unlike the pipette which collects unnecessary bubbles especially when you apply a lot of pressure. The pipette also collects unnecessary blood [volume] while the conical cup is fixed [volume]. The [pipette] flow even if it a bit slow because I experienced it even this morning I was lifting it a bit but blood was not flowing.

**Quote 7: G1:** I don’t want the pipette. When I was doing my work [here and] even at my facility, I was fidgeting [and] I couldn’t get blood to the mark where it is supposed…. otherwise it may even give you wrong results.

**Quote 8: A3:** The pipette is not all that comfortable especially when you are handling malaria RDTs because the problem there is controlling that pressure is not easy. When dispensing there is a tendency of blood dropping before it reaches the right spot

**Quote 9 S1:** I have not been finding difficulties with the conical cup but my work has been easier with the pipette maybe because I have used it for long. When I was trying to use the conical cup, there are moments where some blood still remained in the cup on releasing not like the pipette making work somehow difficult. Otherwise with the pipette, as stated, is soft and then the little blood that you get can be released on the RDT easily. I was feeling some bit of difficulties with the conical cup.

**Quote** **10: A2:** My only observation is that if you don’t fill it well [the conical cup device], there will be a problem touching the spot and blood will not come out. That is the only problem I found but if you master that it is like this it is very easy the moment you touch the spot blood will just flow.

**Quote 11: T1:**  We have been using pipette for a long time but for me I have been experiencing a lot of difficulty. By sucking and releasing, let me say…pressing and releasing……. it is somehow difficult and sometimes you get the blood you put on the testing pad and it may be much then the outcome there if you are not careful, you will not get it proper……..but with the conical cup, I have seen it is very easy because it is just a matter of touching the blood and then it comes up by itself and then just transferring it on the pad. If possible, they can now introduce that…I mean produce that conical cup very much and then they pack it with those RDTs other than the pipettes.

[ ]: added by the authors to facilitate the readers’ understanding

A1, A2,... : identification codes assigned to health workers
